# Supplementary figures and images for: General trends in the calnexin-dependent expression and pharmacological rescue of clinical CFTR variants
Source: eLife. 2025 Dec 11;14:RP107180. doi: 10.7554/eLife.107180 (PMC12698086; doi:10.7554/eLife.107180)

**CANX KO  
HEK293T**

(kDa)

95-

55-

26-

17-

**CANX**

## Cyclophillin B

# B

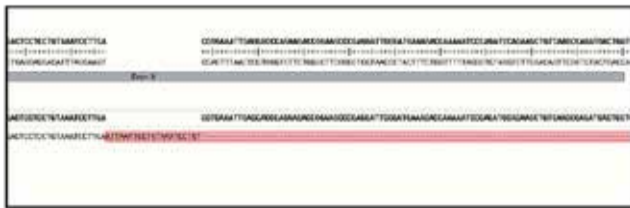

Supplement: Figure 1—source data 1. [file elife-107180-fig1-data1.zip › Figure 1 Supplement 2 Source Data 1/Figure 1 Supplement 2 Source Data 1.pdf]

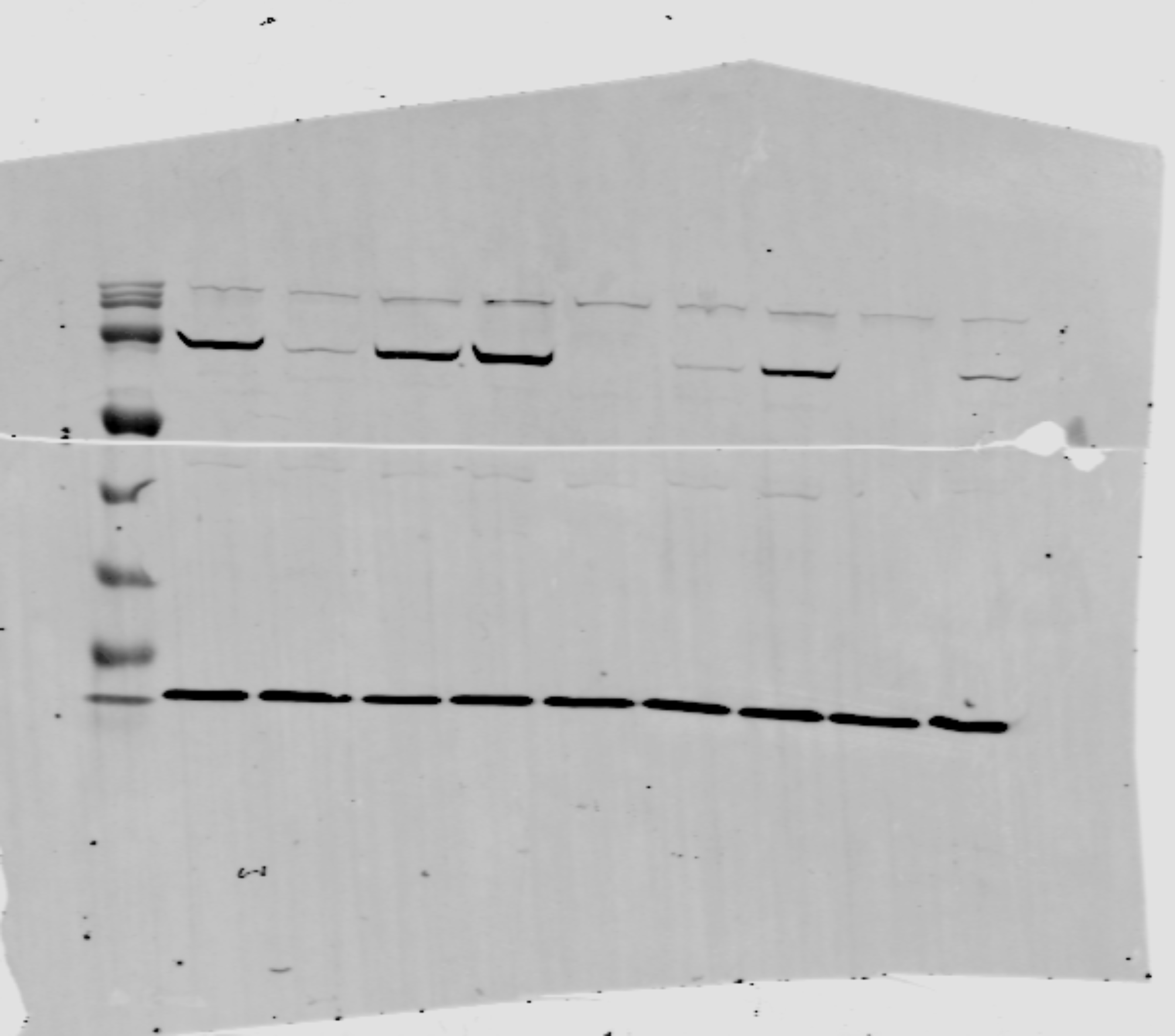

Supplement: Figure 1—source data 2. [file elife-107180-fig1-data2.zip › Figure 1 Supplement 2 Source Data 2/2022-07-23 CRISPR CANX Clone 37 C3 D11 E1 E2 E4 E8 F9 F12.tif]
